# Distribution and density of *Lutraria rhynchaena* Jonas, 1844 relate to sediment while reproduction shows multiple peaks per year in Cat Ba-Ha Long Bay, Vietnam

**DOI:** 10.1515/biol-2020-0072

**Published:** 2020-09-21

**Authors:** Do Manh Hao, Do Trung Sy, Dao Thi Anh Tuyet, Le Minh Hiep, Nguyen Tien Dat, Do Thi Thu Huong, Do Cong Thung, Dang Hoai Nhon, Tran Dinh Lan, Nguyen Van Quan

**Affiliations:** Institute of Marine Environment and Resources, Vietnam Academy of Science and Technology, No. 246 Da Nang Street, Ngo Quyen District, Hai Phong City, Vietnam; Institute of Chemistry, Vietnam Academy of Science and Technology, No. 18 Hoang Quoc Viet Street, Cau Giay District, Ha Noi City, Vietnam; Graduate University of Science and Technology, Vietnam Academy of Science and Technology, No. 18 Hoang Quoc Viet Street, Cau Giay District, Ha Noi City, Vietnam

**Keywords:** *Lutraria rhynchaena*, Cat Ba-Ha Long Bay, distribution, density, reproductive cycle

## Abstract

*Lutraria rhynchaena* Jonas, 1844 is of great commercial interest, but its reserves have dramatically declined over recent decades. Therefore, there is an urgent need of scientific basis to propose effective fishery management measures and improve artificial aquaculture of the clam. In this study, we investigated the distribution and density of *L. rhynchaena*, sediment characteristics, and established the clam’s reproductive cycle through monthly observations from August 2017 to July 2018. The study results showed that distribution and density of clams are related to sediment types, and the sediment type of medium sand is likely the best benthic substrate for the clams. The spawning of clams occurred throughout the year with three spawning peaks in January, April and September. For the sustainable management of the clam resource in Cat Ba-Ha Long Bay, the fishery authorities can issue a ban on harvest of the clam in spawning peak months in January, April and September.

## 1 Introduction

Cat Ba-Ha Long Bay is located on the northeast coast of Vietnam. The bay is characterised by karst towers, many large islands and islets (<3,000). Of which, Cat Ba is the largest island with the area of 298 km^2^. With outstanding universal values, UNESCO recognised Ha Long Bay as World Natural Heritage in 1994 and 2000 and Cat Ba Island as the World Biosphere Reserve in 2014. Karst processes carved hundreds of caves out of the limestone and contributed to the formation of many different unique ecosystems, including lagoons, salt lakes, underground caves, coral reefs, muddy beds, sandy beds, gravel beds and pebble beds. Furthermore, the bay was isolated from mainland for over 18,000 years of its history [1,2]. Therefore, the flora and fauna are very high in biodiversity; many endemic and rare species having universal values are still under conservation and evolution [3]. Among them, *Lutraria rhynchaena* Jonas, 1844 has been recorded to occur only in waters of Cat Ba-Ha Long Bay of Vietnam and a few surrounding countries such as Philippines, Thailand, China and Australia [4,5]. This species is of great commercial interest, but the production has been dramatically declined over recent decades; within 30 years (1979–2008), the reserves of clams fell approximately 100-fold, from about 1.07 to 0.01 ton/ha [4,6]. Although artificial seed has been produced in 2004 [7], artificial cultivation was just bloomed in short time (2004–2011), then rapidly declined due to mass mortality during the rearing cycle. Therefore, there is an urgent need of scientific basis to propose effective fishery management measures and improve artificial aquaculture of the clam.

Area and time restrictions are effective fishery management measures [8]. Of which, area restrictions are to establish area closures whether they are temporary, seasonal or permanent, while time restrictions are to restrict access by fishers to an area in some way. Studies on the biology and ecology of the clams in the area are still scant. In Vietnam, so far, only two studies have been carried out around Cat Ba Island in 1979 and 2008 [4,6]. Therefore, in this study, we focused on two main aims: (1) to investigate the distribution, density and reproductive cycle of *L. rhynchaena* in Cat Ba-Ha Long Bay and (2) to assess their relationship with sediment conditions, temperature and salinity. The timing of clam spawning peaks will be the basis for managers to enact a ban on seasonal or monthly fishing for the clams. The data of distribution and density will be the decisive criteria for the selection of natural clam beds prioritised to protect. For clams that are live buried such as *L. rhynchaena*, sediment provides the clams with refuge to avoid their predators and drifting by water current. The sediment also helps the clams go to the bottom successfully in the D-shaped larval stage [9]. Therefore, the sediment will be one of the most important environmental criteria in selection of clam beds for protection. Moreover, the study results are also useful information in artificial seed production as well as aquaculture of clams.

## 2 Materials and methods

### 2.1 Study site

**Figure 1:**
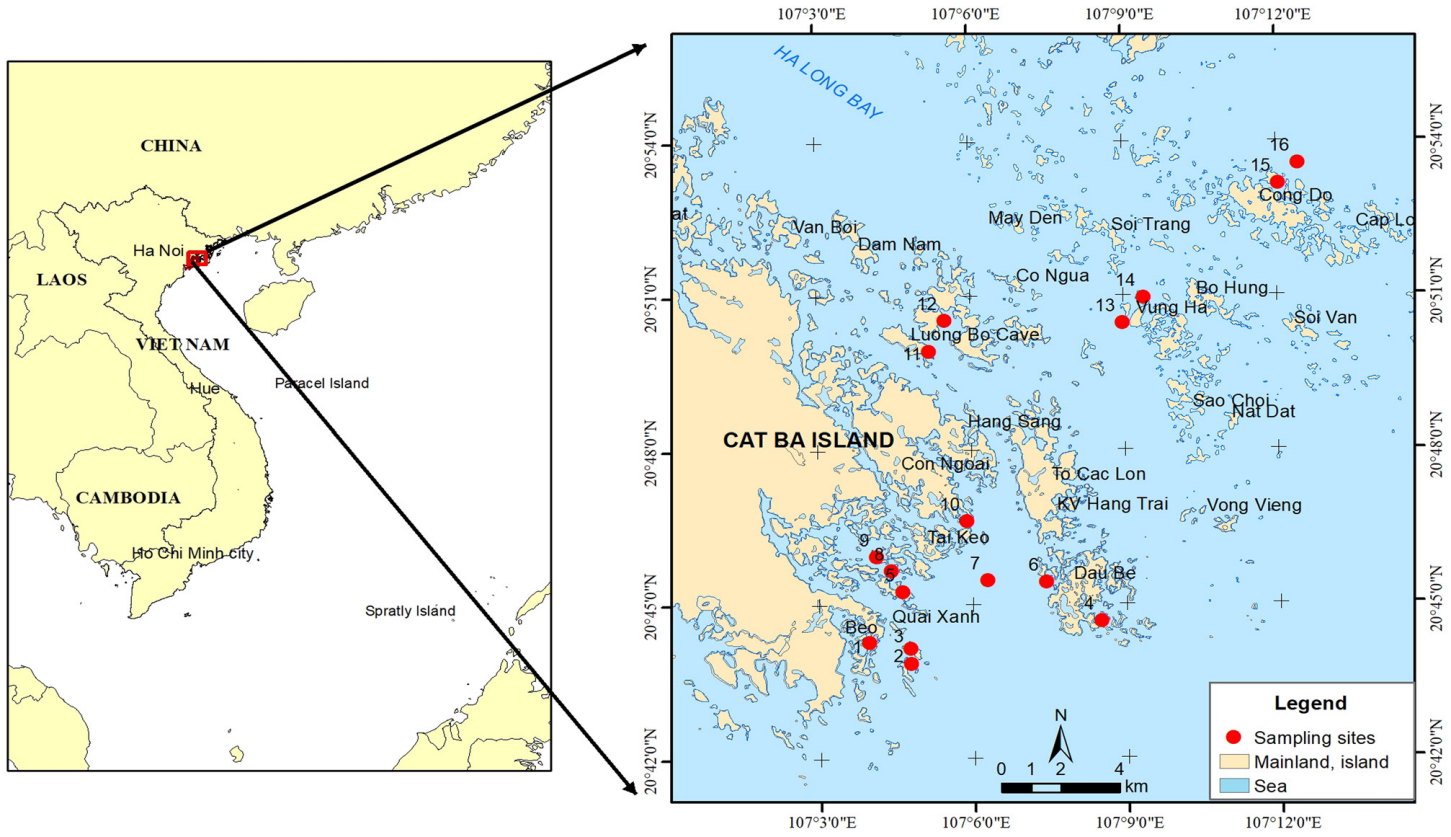
Sampling sites of *L. rhynchaena* Jonas, 1844 in Cat Ba-Ha Long Bay, Vietnam.

The study area was located in the littoral and upper sublittoral zone in Cat Ba-Ha Long Bay (20°43′25.56″N-20°53′22,54″N and 107°3′30,30″E-107°14′10,16″E). In total, 16 sites were chosen to investigate the distribution, density and reproductive cycle of *L. rhynchaena* and measure water temperature, salinity and sediment characteristics between August 2017 and July 2018 (Figure 1). The density of *L. rhynchaena* at each study site was monthly estimated from the average of three plots (5 m × 5 m) which were randomly allocated.

### 2.2 Sampling

*L. rhynchaena* specimens were collected monthly during the daytime when the siphons of the clams were clearly visible. Clams were stored in an icebox for transport and subsequent processing in the laboratory. Surface sediment samples were also collected at the 16 sampling sites using a grab sampler and stored in nylon basket pockets in an icebox until grain-size analysis.

### 2.3 Analysis of environmental variables

The temperature and salinity of surface waters were measured monthly at all sites using a thermometer and a refractometer, respectively. Sediment grain size was analysed using a series of sieves for coarse grain (>63 µm) and the pipette method for fine grain (<63 µm) after removing salts and organic matters with distilled water and 10% hydrogen peroxide. A 63 µm sieve was used to separate coarse from fine grains, and then the sediments were dried at 105°C. Coarse grains were further fractionated using a series of sieves with different pore diameters (i.e. 5,000, 2,500, 2,000, 1,000, 710, 500, 400, 250, 125 and 100 µm) and weighed. To fractionate fine grains, 5 g was dispersed in NaOH and diluted to 1,000 mL with water, stirred, then sampled over time using a 25 mL pipette to obtain fractions at 63, 31, 16, 8, 4, 2 and 1 µm; excess liquid was evaporated, and grains were dried at 105°C before being weighed [10].

All sediment fractions were converted into percent and sediment parameters calculated, including mean diameter (*M*_d_) (1), sorting coefficient (*S*_0_) (2) and skewness (*S*_k_) (3) according to Folk and Ward (1957) and Folk (1980) [11,12]. Sediment types were classified based on Md according to Udden (1914) and Wentworth (1922) [13,14]. All samples and sediment parameters were calculated using the GRADISTAT v8.0 program [15]. Md reflects region condition, especially dynamics (strong, medium or weak conditions). *S*_0_ (very well sorted, well sorted, moderately well sorted, moderately sorted, poorly sorted, very poorly sorted and extremely poorly sorted) and *S*_k_ (very fine skewed, fine skewed, symmetrical, coarse skewed and very coarse skewed) reflect the homogeneity of grain size in sediments under influence of current, wave and tide.

(1) {M}_{\text{d}}=\exp \frac{\mathrm{ln}\hspace{.25em}P16+\hspace{.25em}\mathrm{ln}\hspace{.25em}P50+\hspace{.25em}\mathrm{ln}\hspace{.25em}P84}{3},

(2) {S}_{0}=\exp \left(\frac{\mathrm{ln}\hspace{.25em}P16-\hspace{.25em}\mathrm{ln}\hspace{.25em}P84}{4}+\frac{\mathrm{ln}\hspace{.25em}P5-\hspace{.25em}\mathrm{ln}\hspace{.25em}P95}{6.6}\right),

(3) {S}_{k}=\frac{\mathrm{ln}\hspace{.25em}P16+\hspace{.25em}\mathrm{ln}\hspace{.25em}P84-2(\mathrm{ln}\hspace{.25em}P50)}{2(\mathrm{ln}\hspace{.25em}P84-\hspace{.25em}\mathrm{ln}\hspace{.25em}P16)}+\frac{\mathrm{ln}\hspace{.25em}P5+\hspace{.25em}\mathrm{ln}\hspace{.25em}P95-2(\mathrm{ln}\hspace{.25em}P50)}{2(\mathrm{ln}\hspace{.25em}P95-\hspace{.25em}\mathrm{ln}\hspace{.25em}P5)},

where *P*5, *P*16, *P*50, *P*84 and *P*95 is grain diameter at 5%, 16%, 50%, 84% and 95% at the cumulative percentile value, respectively.

### 2.4 Histological techniques

A total of 401 individuals of *L. rhynchaena* were brought to the laboratory where the visceral mass was removed and fixed in Bouin’s solution for 24 h, then preserved in 70% ethanol. Samples were initially sectioned into slices less than 28 mm thick, dehydrated in 95% ethanol for 4 h, softened in methyl salicylate for 12–24 h, embedded in melting paraffin at 65°C for at least 6 h, sectioned by a microtome into slices of 5–7 µm thick and fixed on glass microscope slides before staining with Hematoxylin Harris for 10 min, rinsed with distilled water for 5 min, then stained with 1% Eosin Y for 12 min. The prepared slides were examined and photographed using a light microscope with attached camera at 400× magnification to determine sex and reproductive stage.

The presence of seminal vesicles containing hair-like spermatozoa in the gonad indicated a male clam, while the presence of follicles with oogonia or polygonal-shaped oocytes indicated a female clam. For hermaphroditic individuals, there were both hair-like spermatozoa and oogonia in the gonad.

Clam reproductive maturity was categorised into five stages using the maturity scale described by Cross et al. (2012) [16], whereby maturity stages were designated as “indifferent, developing, ripe, spawning and spent”. When more than one developmental stage was present in a single individual, maturity was scored based on which stage accounts the highest percentage.

### 2.5 Female gonad development

The indifferent stage of development of the female reproductive system was determined when specific inclusions were seen in the follicle cells, and early oocytes were present in the alveolar membrane. The developing stage is recognised by an increasing number and size of oocytes. There is also a central lumen in each follicle into which stalked oocytes protrude. At the ripe stage of development in the female reproductive system, many mature, spherical free oocytes are found within the follicular lumen. At the spawning stage, the number of oocytes decreases and empty spaces in the lumen appear as a result of the release of mature oocytes. Emptying follicles and the cessation of oogenesis in all follicles are characteristics of this stage. At the last stage, the gonads are collapsed and irregular in shape, with big empty spaces in the lumen and few germinal cells.

### 2.6 Male gonad development

The indifferent stage of development of the male reproductive system is characterised by the presence of aberrant forms of multinucleated non-pyctonic and pyctonic cells. The basal membrane and follicle cells are the dominant structural elements, with a few primary spermatocytes or spermatogonia visible at the periphery of the lumen. In the developing stage, the maturation and proliferation of the spermatocytes take place. In ripe male clams, the follicle is filled with dense radiating bands of spermatozoa with tails which project into the central lumen. At the spawning stage, the follicle is characterised by lower number of spermatozoa compared to the ripe stage. A few spermatozoa remain in the radiating bands, but the rows of follicle cells gradually increase to replace the spawned spermatozoa. At the spent stage in males, the follicles are almost completely filled with follicle cells, and the reduced lumen contains a few sex cells.

### 2.7 Statistical analysis

The sex ratio of clams was calculated by dividing the number of female individuals by the number of male individuals. A Chi-squared test was used to determine whether there was a significant difference between the observed female:male sex ratio and the expected female:male sex ratio of 1:1. Single-factor analysis of variance (ANOVA) was used to compare the mean values of temperature and salinity temporally and spatially and examine the effects of location or sediment types on the density of clam with a significance level of 0.05. If the ANOVA was significant, the post-hoc tests were further conducted to compare the difference in mean values between each pair of samples. *F*-Test Two-Sample for Variances used to examine the variance equal between each pair of samples before *T*-Test Two-Sample Assuming Equal Variances or *T*-Test Two-Sample Assuming Unequal Variances was used to compare the difference in mean values of clam’s density between each pair of samples (sampling sites and sediment types). *T*-Test paired two sample for means was used to compare the difference in mean values of temperature and salinity spatially and temporally.

## 3 Results

### 3.1 Physicochemical factors

#### 3.1.1 Water temperature and salinity

**Figure 2:**
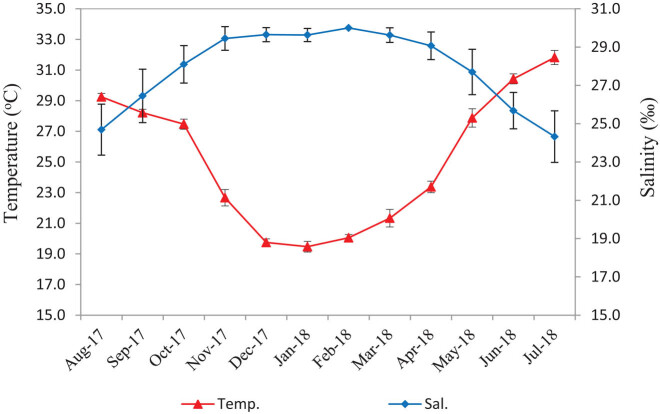
Monthly variations of surface water temperature and salinity at study sites.

During the study period, the average water temperature ranged from 19.0 to 32.5°C, with a minimum of 19.0°C recorded in January 2018 and a maximum of 32.5°C in July 2018 (Figure 2). There was no significant difference of temperature spatially, but there was significant difference in monthly temperature (df = 15, *α* = 0.05). However, there were no significant differences in temperature between May (2018) and September (2017) (*p* = 0.04, *α* = 0.01), and between May (2018) and October (2017) (*p* = 0.04, *α* = 0.01). The temperature in January was significantly the lowest than those of other months (*p* ≤ 2.4 × 10^−4^, *α* = 0.01) (Table A1). The salinity was around 28.0–30.0‰ in all of the studied sites during dry months (November–March) but decreased to 22.0‰ at site No. 01 near the coast during the rainy season (May–September) due to river discharge and tidal effects (Figure 2). There was no significant difference of salinity spatially (df = 15, *α* = 0.05), but there was significant difference in salinity seasonally (df = 11, *α* = 0.05). However, there were no significant differences in salinity among November, 2017 (Nov-17), December, 2017 (Dec-17), January, 2018 (Jan-18) and March, 2018 (Mar-18) (df = 3, *α* = 0.01), and between October, 2017 (Oct-17) and May, 2018 (May-18) (*p* = 0.03, *α* = 0.01) (Table A2).

### 3.2 Sedimentary environment

The sedimentary environment consisted of submerged plain, coral reefs and tidal channels. Submerged plain occupied the largest area of the whole Cat Ba-Ha Long Bay region surveyed. Water depth was generally 2–5 m but reached 20 m in the areas of Dau Be and Lan Ha. The benthos was mainly covered by fine silt, and rarely by sand and shell debris.

Based on the grain size characteristics and covering of living coral, the sediments were separated into seven different sediment types, consisting of very coarse sand, very coarse sand + coral reef, coarse sand, coarse sand + coral reef, medium sand, coarse silt and fine silt. The very coarse sands had the *M*_d_ of 1176.7 µm, consisting of clastic particles and shell debris. They were very poorly sorted, and the sediment is towards small particles. Very coarse sand + coral reef had Md ranging from 1154.5 to 1560.9 µm, consisting of clastic particles, shell debris, living coral and rock bottom. They were very poorly to poorly sorted, and the sediment was towards small particles and symmetrical. The coarse sands had *M*_d_ ranging from 581.5 to 757.8 µm, consisting of dead coral particles and shell debris. The coarse sand + coral reef had an *M*_d_ of 665.6 µm, consisting of sand, shell debris, living coral and rock bottom. Both the coarse sand with and without coral reef were poorly sorted and towards fine skewed and symmetrical. The medium sand had *M*_d_ ranges from 399.4 to 491.5 µm, was very poorly to poorly sorted and the sediment towards very coarse skewed and fine skewed. The coarse silt had an *M*_d_ of 21.4 µm, was very poorly sorted and skewed towards very fine. Fine silt had an *M*_d_ of 6.0 µm and was very poorly sorted (Table 1).

**Table 1:** Characteristics of sediment distribution and density of *L. rhynchaena* in Cat Ba-Ha Long Bay

Sites	*M*_d_ (µm)	*S*_0_	*S*_k_	Sediment types	Sorting	Skewness	Density (Ind./100 m^2^)
1	708.10	2.98	−0.15	Coarse sand	Poorly sorted	Fine skewed	6.9
2	581.50	3.92	−0.24	Coarse sand	Poorly sorted	Fine skewed	26.3
3	6.30	6.03	0.14	Fine silt	Very poorly sorted	Coarse skewed	0.0
4	1560.80	2.67	−0.03	Very coarse sand + coral reef	Poorly sorted	Symmetrical	22.2
5	399.40	3.77	0.36	Medium sand	Poorly sorted	Very coarse skewed	30.8
6	1560.90	2.67	−0.04	Very coarse sand + coral reef	Poorly sorted	Symmetrical	24.7
7	6.40	6.01	0.14	Fine silt	Very poorly sorted	Coarse skewed	0.0
8	757.80	2.84	−0.16	Coarse sand	Poorly sorted	Fine skewed	58.9
9	6.30	6.02	0.14	Fine silt	Very poorly sorted	Coarse skewed	0.0
10	665.60	3.41	−0.07	Coarse sand + coral reef	Poorly sorted	Symmetrical	10.7
11	21.40	6.13	−0.70	Coarse silt	Very poorly sorted	Very fine skewed	0.0
12	491.50	6.00	−0.25	Medium sand	Very poorly sorted	Fine skewed	94.7
13	21.40	6.14	−0.70	Coarse silt	Very poorly sorted	Very fine skewed	0.0
14	1176.70	4.77	−0.33	Very coarse sand	Very poorly sorted	Very fine skewed	33.2
15	1154.50	4.95	−0.37	Very coarse sand + coral reef	Very poorly sorted	Very fine skewed	28.0
16	5.20	5.11	0.16	Fine silt	Very poorly sorted	Symmetrical	0.0

### 3.3 The distribution and density

*L. rhynchaena* was found to be only distributed in small beds scattered around islands. The presence of clams tended to depend on substrate compositions, usually occurring on five different substrate types: very coarse sand + coral reef, very coarse sand, coarse sand + coral reef, coarse sand and medium sand. We did not find the clam in other substrate types such as coarse silt or fine silt, although these substrate types dominated the seabed in Cat Ba-Ha Long Bay. The clam occurs in environments where salinity ranged from 22.0 to 30.0‰. Salinity in the area fluctuates seasonally, with some coastal sites being characterised by low salinity during the rainy season (Figure 2).

The population density of the clam was low in almost all investigated sites, ranging from 0.0 to 94.7 ind./100 m^2^ (Table 1). Based on sampling locations, the density of clam was categorised into six significant different groups (df ≥ 35, *α* = 0.01) (Table 2). The first density group was the highest one −94.7 ± 31.2 Ind./100 m^2^ (*n* = 36) at site No. 12. The second density group was 58.9 ± 25.6 Ind./100 m^2^ (*n* = 216) at site No. 08. The third density group ranged from 27.5 ± 11.9 Ind./100 m^2^ (*n* = 36) at sites: No. 02, 04, 05, 06, 14 and 15. The fourth density group was 10.7 ± 2.3 Ind./100 m^2^ (*n* = 36) at site No. 10. The fifth density group was 6.9 ± 6.0 Ind./100 m^2^ (*n* = 36) at site No. 1. The sixth density group was 0.0 Ind./100 m^2^ (*n* = 216) at sites: No. 03, 07, 09, 11, 13 and 16.

**Table 2:** *t*- and *p*-values between each pair of density groups of *L. rhynchaena* based on sampling locations using *t*-test (*α* = 0.01)

*t*-Test	First group	Second group	Third group	Fourth group	Fifth group	Sixth group
First group						
Second group	5.3/1.2 × 10^−06^					
Third group	12.7/4.2 × 10^−15^	7.2/1.2 × 10^−08^				
Fourth group	16.1/9.5 × 10^−18^	11.3/2.3 × 10^−13^	18.8/1.9 × 10^−49^			
Fifth group	16.6/5.7 × 10^−19^	11.9/1.6 × 10^−14^	16.0/5.5 × 10^−28^	3.5/1.0 × 10^−03^		
Sixth group	18.2/2.0 × 10^−19^	13.8/9.6 × 10^−16^	34.1/1.1 × 10^−88^	27.3/3.4 × 10^−25^	6.9/5.6 × 10^−08^	

**Figure 3:**
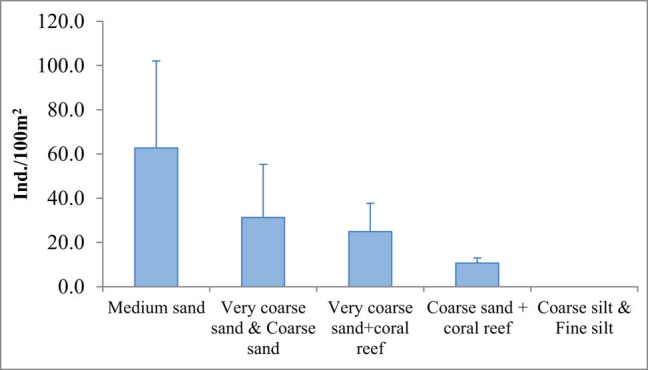
Density of *L. rhynchaena* as a function of sediment type in the surveys (2017–2018). Error bars present standard deviation.

Based on sediment types, the density of clam was categorised into five significant different density groups (df ≥ 35, *α* = 0.01) (Table 3). The first group has the highest density of 62.7 ± 39.4−Ind./100 m^2^ (*n* = 72) in medium sand beds. The second group has the density ranging from 31.3 ± 23.9 Ind./100 m^2^ (*n* = 144) in very coarse sand beds and coarse sand beds. The third group has density of 25.0 ± 12.8 Ind./100 m^2^ (*n* = 108) in very coarse sand + coral reef bed. The fourth group has density of 10.7 ± 2.3 Ind./100 m^2^ (*n* = 36) in coarse sand + coral reef bed. The fifth group has density of 0.0 Ind./100 m^2^ (*n* = 108) in coarse silt beds and fine silt beds (Figure 3).

**Table 3:** *t*- and *p*-values between each pair of density groups of *L. rhynchaena* based on sediment types using *t*-test (*α* = 0.01)

*t*-Test	First group	Second group	Third group	Fourth group	Fifth group
First group					
Second group	6.2/1.3 × 10^−08^				
Third group	7.9/1.4 × 10^−11^	2.7/7.0 × 10^−03^			
Fourth group	11.2/2.1 × 10^−17^	10.2/7.1 × 10^−19^	11.0/3.0 × 10^−20^		
Fifth group	13.5/2.6 × 10^−21^	15.7/6.2 × 10^−33^	20.2/2.0 × 10^−38^	27.3/3.0 × 10^−25^	

### 3.4 Sex ratio

**Figure 4:**
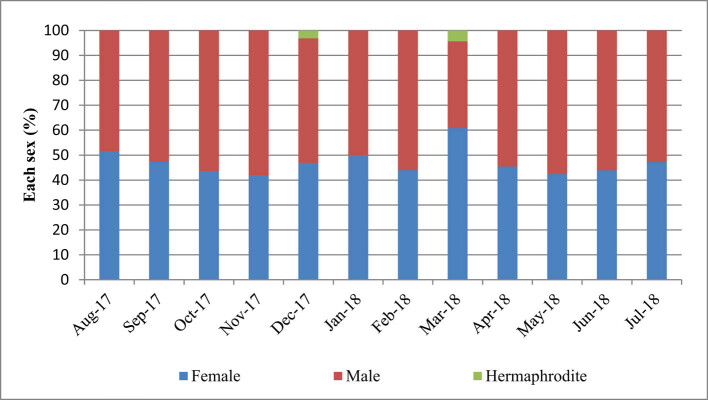
Sex ratio of *L. rhynchaena* at Cat Ba-Ha Long Bay over the study period.

A total of 401 individuals of *L. rhynchaena* were collected over 12 months from August 2017 to July 2018. The results revealed that 188 (46.88%) were female, 211 (52.62%) were male and two (0.50%) were determined as hermaphrodites. The overall female:male sex ratio was 1:1.12. However, Chi-square test showed that the ratio was not significantly different from a 1:1 ratio (*χ* ^2^ = 1.4, df = 1, *p* > 0.05). Although statistical tests were not applied to individual months, all months were close to a 1:1 sex ratio with slightly more males than females, with the exception of August and March when there were more females (Figure 4).

### 3.5 Reproductive cycle

#### 3.5.1 Females

**Figure 5:**
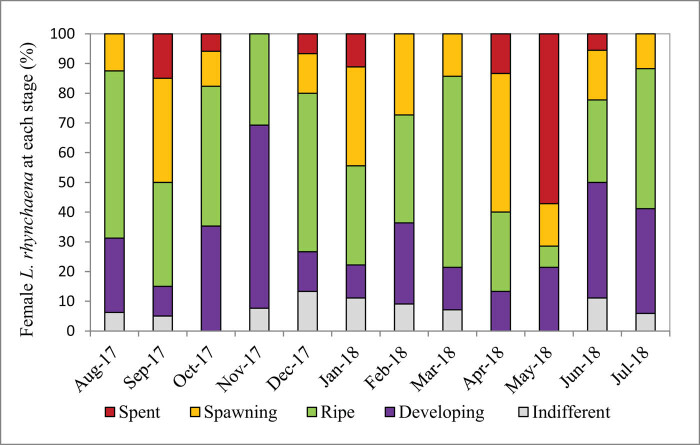
Stages of gametogenesis observed in female clams over the study period.

**Figure 6:**
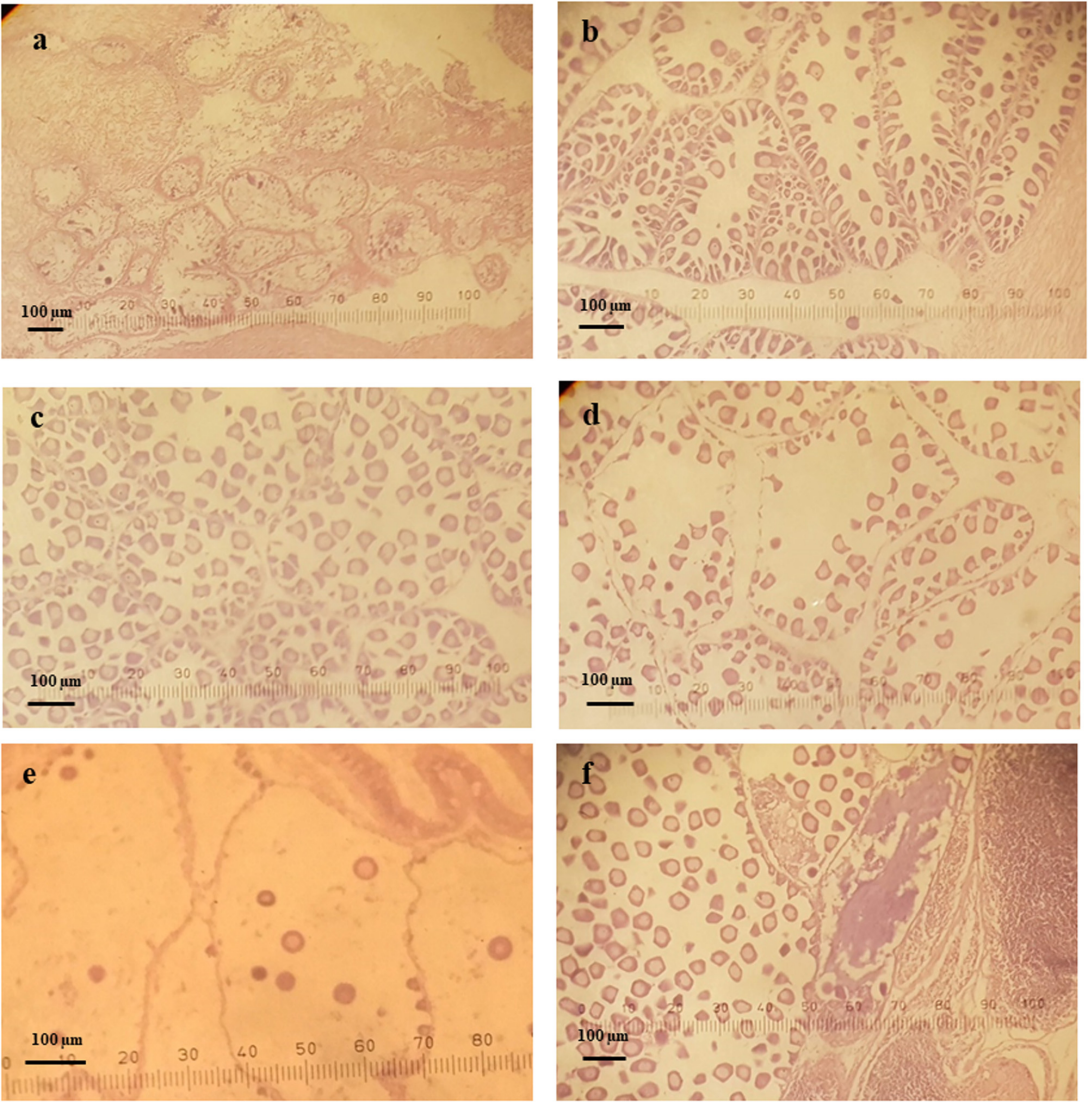
Microphotographs of gonadal development stages of female *L. rhynchaena*: (a) indifferent, (b) developing, (c) ripe, (d) spawning, (e) spent and (f) hermaphrodite.

During the course of the study, all five stages of gametogenesis were observed in *L. rhynchaena*. The percentage of clams at the indifferent stage ranged from 0.00 to 13.33%, and there were no clams at this stage of gametogenesis in April, May or October. The highest percentages of clams at the indifferent stage were recorded in December (13.33%), January (11.11%) and June (11.11%). The percentage of clams at the developing stage ranged from 10.00% to 61.54%, with the lowest percentages being recorded in January (11.11%), April (13.33%) and September (10.00%). The highest percentages at this stage were recorded in November (61.54%), June (38.89%) and October (35.29%). The percentage of clams at the ripe stage ranged from 7.14% to 64.29%, with an average value of 38.75%. The highest percentages were recorded in March (64.29%), August (56.25%) and December (53.33%), whereas the lowest percentage was recorded in May (7.14%). The percentage of clams at the spawning stage ranged from 0.00 to 46.67%, with an average value of 19.74%. The highest percentages were recorded in April (46.67%), September (35.00%) and January (33.33%). In November, spawning clams were not found. The percentage of clams at the spent stage ranged from 0.00 to 57.14%, with an average value of 9.56%, and the highest percentage was recorded in May (57.14%) (Figures 5 and 6).

### 3.6 Males

**Figure 7:**
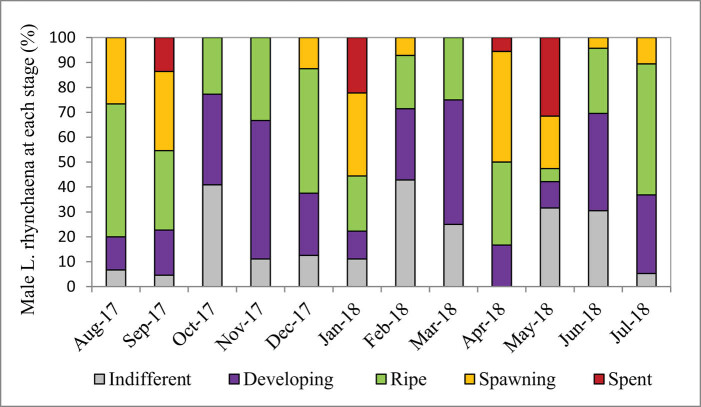
Stages of gametogenesis observed in male clams over the study period.

**Figure 8:**
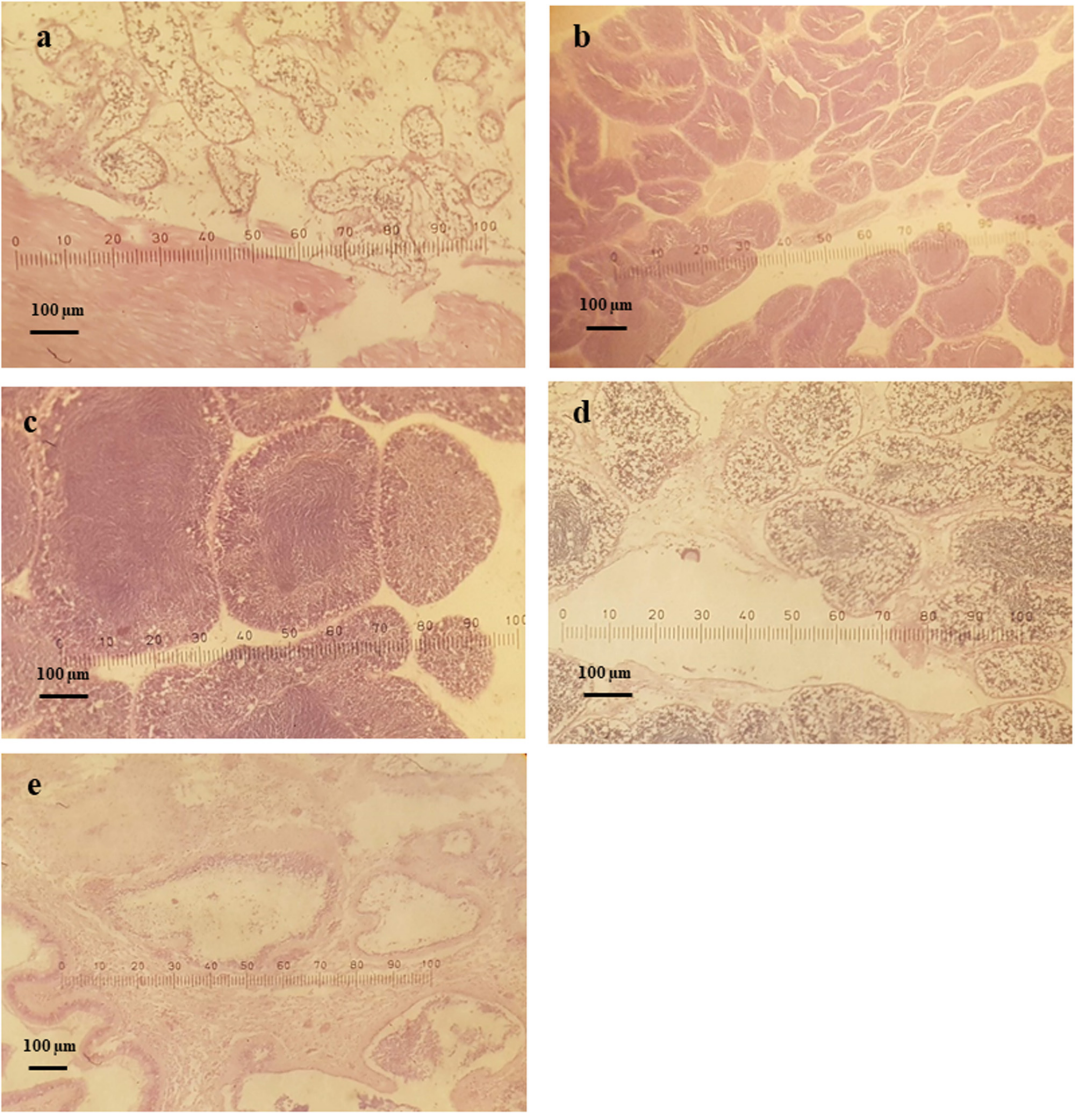
Microphotographs of gonadal development stages of male *L. rhynchaena*: (a) indifferent, (b) developing, (c) ripe, (d) spawning and (e) spent.

The average percentage of male clams at the ripe stage was highest (31.43%), followed by the developing stage (28.00%), indeterminate stage (18.50%) and spawning stage (15.99%). The percentage of clam at the spent stage was lowest (6.08%). The percentage of clams at the indifferent stage ranged from 0.00 to 42.86%, with the highest percentages being recorded in February (42.86%), October (40.91%), May (31.58%) and June (30.43%); in the remaining months, the percentage was lower than 25.00%. The percentage of clams at the developing stage ranged from 10.53% to 55.56%, with the highest percentages being recorded in November (55.56%) and March (50.00%). The percentage of clams at the ripe stage ranged from 5.26% to 53.30%. The highest percentages were recorded in August (53.33%), December (50.00%) and July (52.63%), whereas the lowest percentages of clams at this ripe stage were registered in May (5.26%), February (21.43%) and October (22.73%). The percentage of clams at the spawning stage ranged from 0.00 to 44.44%, and we failed to find any clams at this stage in October, November or March; in February and July, the percentage of spawning stage clams was low (4.35–7.14%). The highest percentages of clams at the spawning stage were recorded in April (44.44%), January (33.33%) and September (31.82%). The percentage of clams at the spent stage ranged from 0.00 to 31.58%, and we failed to find any clams at this stage in all months with the exception of January (22.22%), April (5.56%), May (31.58%) and September (13.64%) (Figures 7 and 8).

## 4 Discussion

### 4.1 Sediment types, distribution and density of *L. rhynchaena*

This study revealed that the distribution of *L. rhynchaena* was associated with substrate types in Cat Ba-Ha Long Bay. The clam also inhabited within rock slits on hard bottom, with or without coral covering. On the soft bottom, the clams were only distributed on the following substrate types: sand, coral and shell debris. The clams were not found on coarse silt or fine silt substrate types, although these are the dominant substrate types within the study area. Substrate type influences the distribution of macro-benthic communities [17,18,19,20,21,22,23]. For example, *Mya arenaria* with burrowing behaviour similar to *L. rhynchaena* primarily inhabits sand and mud substrates [24], with higher densities being observed in sand substrates [25]. In some cases, growth rates of clams can be impacted by sediment type, with slower growth rates being associated with the sites comprising greater contents of fine particle (silt and clay) sediments [20]. Certain substrate compositions provide bivalves with refuge against predators. Sediment type can also influence anti-predator abilities of clams, and although coarse sediments have the most energy consumption for metabolic activities, they have lower predation risk compared to finer sediments [25]. Moreover, differences in clam densities between sediment types could also be related to variations in feeding conditions, which were probably determined by localised features such as the hydrodynamic regime [20]. The study results showed that the density of *L. rhynchaena* is significantly related to benthic substrate types; the natural clam beds with medium sands could be the best substrate environments for the clam.

### 4.2 Gametogenesis development and environmental conditions

The female:male sex ratio of *L. rhynchaena* in the present study was approximately 1:1.12, slightly more in favour of males but the difference is not significant. The trend that the percentage of male individuals is higher than that of females was previously observed for *L. rhynchaena* in North Bais Bay, Philippines [5], and in other clam species as well [16,26,27]. We registered two hermaphroditic clams, which is the first evidence of the phenomenon for *L. rhynchaena*. However, the hermaphroditism has been described in several other clam species [27]. Although self-fertilisation is not possible, this characteristic allows them to reproduce with both male and female clams. This reduces the burden of finding a compatible mate, while simultaneously doubling the number of offspring produced by the process. As with all other forms of sexual reproduction, hermaphroditism ensures that new gene combinations be passed on to further generations [28]. The reproductive strategy may be the adaptation of the clam with sparse density condition. Although the protandry of *L. rhynchaena* was found in North Bais Bay, Philippines [5], it was not found in Cat Ba-Ha Long Bay. The result showed that the reproductive characteristic of *L. rhynchaena* could change according to locally environmental conditions.

All five stages of gametogenesis were observed in *L. rhynchaena* over the period of sampling. The presence of gonads in the developing, ripe and spawning stages during most months of the year may suggest that gametogenesis, maturity and spawning of *L. rhynchaena* occur throughout the year. We observed three peaks of the spawning stage in female clams in September (2017), January (2018) and April (2018). The highest spawning stage peak (46.67%) was in April (2018), which was followed by a very high percentage (57.14%) of spent clams in May (2018), revealing a key spawning event. In January (2018), we have also observed a high percentage of both spawning and spent clams (33.33% and 11.11%, respectively), but there were not any clams in the spent stage in the subsequent month. In September (2017), we have observed a similar trend of gametogenesis development as in January. According to Bantoto and Ilano (2011) [5], *L. rhynchaena* also has gonad in developing, mature and spawning stages occurring throughout the year, with two spawning peaks in January and June. The divergence of spawning time was also recorded for other clam species (e.g. *Mya arenaria* and *Marcia optima*). In Skagit Bay, Washington, USA, spawning of *Mya arenaria* lasts from late May to early August, with its peak in June–July [26]. In Cape Ann, Massachusetts, clams have been recorded to spawn twice each year, in March–April and June–July [16]. In eastern Atlantic waters, the majority of spawning periods has been recorded in the summer months, May and June [16]. On the coast of India, the spawning season of *Marcia optima* has been recorded from May to July in the south-east, while in the south-west, it is from March to May [29].

It is noteworthy that the percentage of clams in the ripe stage of maturity was often high throughout the year, with the exception of the month following the peak-spawning month. It seems that gametogenesis of clams was affected by local environmental conditions. In temperate waters, rising temperatures in summer months can synchronise the ripe and spawning stages of bivalve molluscs [16,30,31,32,33,34,35,36]. According to Nelson (1928) [32], spawning of bivalve molluscs occurs on a rising temperature, but maturation progress is triggered at a temperature slightly below that in which spawning occurs. Temperature also appears to be a more critical factor in the timing of gonad maturation than in triggering the release of gametes in *Mya arenaria* [30]. Spawning of *Mya arenaria* peaks in the months after water temperatures reaches its highest [16]. Temperature also probably controls gonad development of the venerid clam *Meretrix lusoria* in Ariake Sound and Tokyo Bay in Japan [31]. In tropical waters, the change of water temperature influences the spawning of clam species [5,27]. According to Bantoto and Ilano (2011) [5], the increase and decrease of temperature over the seasons could have triggered spawning events they observed in *L. rhynchaena* in the Philippines. An increase of 5°C of water temperature, from 27°C in January to 32°C in June, could have triggered the spawning of the clam which peaked in June. Similarly, the decrease of 3.1°C from 32°C in June to 28.9°C in December might make it possible for gonads to mature, resulting in another spawning peak in January. The study results of Hwang (2007) [27] showed that both the pearl oysters *Pinctada fucata* and *Pinctada margaritifera* exhibited different annual cyclical patterns, in which maturity peaked in May and October for *P. fucata*, and in July and November for *P. margaritifera*. Spawning peaks of *P. fucata* were synchronised with rising and decreasing temperatures in May and October. In the current study, *L. rhynchaena* had three spawning peaks in April, September and January, with the highest peak in April. With respect to temperatures, only the spawning peak in January is synchronised with the “critical” temperature, the lowest one; the two remaining spawning peaks occurred during rising and decreasing temperature events. However, April and October are transition months of the northern monsoon and southwest’s wind. The northern monsoon lasts from November to March, and the southwest’s wind lasts from May to September. During the northern monsoon, the climate is cold with less rain, but during periods with southwesterly winds, the climate is hot and rains heavily. Seasonal temperature changes likely trigger maturation and spawning of *L. rhynchaena* in Cat Ba-Ha Long Bay. For the sustainable management of the clam resource in Cat Ba-Ha Long Bay, the fishery authorities can issue a ban on harvest of the clam in spawning peak months in January, April and September.

## Appendix

**Table A1:** *t*- and *p*-values between each pair of monthly temperature based on monitoring result in Cat Ba-Ha Long Bay during 2017–2018 using *t*-test (*α* = 0.01)

	Aug-17	Sep-17	Oct-17	Nov-17	Dec-17	Jan-18	Feb-18	Mar-18	Apr-18	May-18	Jun-18	Jul-18
Aug-17												
Sep-17	14/5 × 10^−10^											
Oct-17	17/5 × 10^−11^	8/2 × 10^−06^										
Nov-17	41/8 × 10^−17^	33/2 × 10^−15^	38/3 × 10^−16^									
Dec-17	120/9 × 10^−24^	81/3 × 10^−21^	85/1 × 10^−21^	26/6 × 10^−14^								
Jan-18	82/3 × 10^−21^	74/1 × 10^−20^	71/2 × 10^−20^	33/2 × 10^−15^	5/2 × 10^−04^							
Feb-18	127/4 × 10^−24^	103/8 × 10^−23^	58/4 × 10^−19^	17/5 × 10^−11^	−3/5 × 10^−03^	−5/10 × 10^−05^						
Mar-18	46/1 × 10^−17^	43/4 × 10^−17^	31/4 × 10^−15^	7/3 × 10^−06^	−10/3 × 10^−08^	−14/5 × 10^−10^	−10/4 × 10^−08^					
Apr-18	47/1 × 10^−17^	40/1 × 10^−16^	32/4 × 10^−15^	−6/5 × 10^−05^	−43/4 × 10^−17^	−60/3 × 10^−19^	−31/6 × 10^−15^	−24/2 × 10^−13^				
May-18	10/6 × 10^−08^	2/4 × 10^−02^	−2/4 × 10^−02^	−20/2 × 10^−12^	−43/4 × 10^−17^	−37/4 × 10^−16^	−52/3 × 10^−18^	−25/1 × 10^−13^	−19/6 × 10^−12^			
Jun-18	−10/6 × 10^−08^	−21/2 × 10^−12^	−24/3 × 10^−13^	−58/4 × 10^−19^	−137/1 × 10^−24^	−154/2 × 10^−25^	−100/1 × 10^−22^	−65/8 × 10^−20^	−82/2 × 10^−21^	−13/2 × 10^−09^		
Jul-18	−22/8 × 10^−13^	−27/3 × 10^−14^	−66/8 × 10^−20^	−72/2 × 10^−20^	−125/5 × 10^−24^	−99/2 × 10^−22^	−76/8 × 10^−21^	−46/2 × 10^−17^	−54/2 × 10^−18^	−19/5 × 10^−12^	−9/1 × 10^−07^	

**Table A2:** *t-* and *p*-values between each pair of monthly salinity based on the monitoring result in Cat Ba-Ha Long Bay during 2017–2018 using *t*-test (*α* = 0.01)

	Aug-17	Sep-17	Oct-17	Nov-17	Dec-17	Jan-18	Feb-18	Mar-18	Apr-18	May-18	Jun-18	Jul-18
Aug-17												
Sep-17	−20/3 × 10^−12^											
Oct-17	−21/1 × 10^−12^	−11/1 × 10^−08^										
Nov-17	−23/4 × 10^−13^	−14/7 × 10^−10^	−11/2 × 10^−08^									
Dec-17	−19/9 × 10^−12^	−11/1 × 10^−08^	−9/2 × 10^−07^	−2/2 × 10^−02^								
Jan-18	−16/6 × 10^−11^	−10/4 × 10^−08^	−8/2 × 10^−06^	−2/1 × 10^−01^	0/8 × 10^−01^							
Feb-18	−16/8 × 10^−11^	−10/4 × 10^−08^	−8/1 × 10^−06^	−4/3 × 10^−03^	−4/2 × 10^−03^	−4/7 × 10^−04^						
Mar-18	−20/4 × 10^−12^	−12/5 × 10^−09^	−10/8 × 10^−08^	4/2 × 10^−02^	1/6 × 10^−01^	0/9 × 10^−01^	4/1 × 10^−03^					
Apr-18	−26/8 × 10^−14^	−14/6 × 10^−10^	−7/8 × 10^−06^	−7/2 × 10^−03^	4/5 × 10^−04^	3/4 × 10^−03^	5/1 × 10^−04^	5/3 × 10^−04^				
May-18	−31/4 × 10^−15^	−11/1 × 10^−08^	2/3 × 10^−02^	9/2 × 10^−07^	8/9 × 10^−07^	7/5 × 10^−06^	8/1 × 10^−06^	9/4 × 10^−07^	11/2 × 10^−08^			
Jun-18	−5/1 × 10^−04^	4/2 × 10^−03^	27/5 × 10^−14^	33/2 × 10^−15^	24/3 × 10^−13^	21/1 × 10^−12^	18/1 × 10^−11^	27/5 × 10^−14^	19/5 × 10^−12^	10/5 × 10^−08^		
Jul-18	5/3 × 10^−04^	19/6 × 10^−12^	26/8 × 10^−14^	25/1 × 10^−13^	20/2 × 10^−12^	18/2 × 10^−11^	17/4 × 10^−11^	21/1 × 10^−12^	26/9 × 10^−14^	25/1 × 10^−13^	8/2 × 10^−06^	
